# Study protocol for a randomized, controlled, superiority trial comparing the clinical and cost- effectiveness of integrated online mental health assessment-referral-care in pregnancy to usual prenatal care on prenatal and postnatal mental health and infant health and development: the Integrated Maternal Psychosocial Assessment to Care Trial (IMPACT)

**DOI:** 10.1186/1745-6215-15-72

**Published:** 2014-03-06

**Authors:** Dawn Kingston, Marie-Paule Austin, Kathy Hegadoren, Sheila McDonald, Gerri Lasiuk, Sarah McDonald, Maureen Heaman, Anne Biringer, Wendy Sword, Rebecca Giallo, Tejal Patel, Marie Lane-Smith, Sander Veldhuyzen van Zanten

**Affiliations:** 1 University of Alberta, 11405-87th Avenue, Edmonton T6G 1C9 AB, Canada; 2 University of New South Wales, Sydney, Australia; 3 University of Calgary, Calgary, Canada; 4 McMaster University, Hamilton, Canada; 5 University of Manitoba, Winnipeg, Canada; 6 University of Toronto, Toronto, Canada; 7 Murdoch Children’s Research Institute, Melbourne, Australia

**Keywords:** psychosocial assessment, online, screening, cognitive behavior therapy, pregnancy, depression, anxiety, stress, randomized controlled trial

## Abstract

**Background:**

Stress, depression, and anxiety affect 15 to 25% of pregnant women. However, fewer than 20% of prenatal care providers assess and treat mental health problems and fewer than 20% of pregnant women seek mental healthcare. For those who seek treatment, the lack of health system integration and existing barriers frequently prevent treatment access. Without treatment, poor prenatal mental health can persist for years and impact future maternal, child, and family well-being.

**Methods/Design:**

The purpose of this randomized controlled trial is to evaluate the effectiveness of an integrated process of online psychosocial assessment, referral, and cognitive behavior therapy (CBT) for pregnant women compared to usual prenatal care (no formal screening or specialized care). The primary outcome is self-reported prenatal depression, anxiety, and stress symptoms at 6 to 8 weeks postrandomization. Secondary outcomes are postpartum depression, anxiety, and stress symptoms; self-efficacy; mastery; self-esteem; sleep; relationship quality; coping; resilience; Apgar score; gestational age; birth weight; maternal-infant attachment; infant behavior and development; parenting stress/competence; and intervention cost-effectiveness, efficiency, feasibility, and acceptability. Pregnant women are eligible if they: 1) are <28 weeks gestation; 2) speak/read English; 3) are willing to complete email questionnaires; 4) have no, low, or moderate psychosocial risk on screening at recruitment; and 5) are eligible for CBT. A sample of 816 women will be recruited from large, urban primary care clinics and allocation is by computer-generated randomization. Women in the intervention group will complete an online psychosocial assessment, and those with mild or moderate depression, anxiety, or stress symptoms then complete six interactive cognitive behavior therapy modules. All women will complete email questionnaires at 6 to 8 weeks postrandomization and at 3, 6, and 12 months postpartum. Clinic-based providers and researchers conducting chart abstraction and analysis are blinded. Qualitative interviews with 8 to 10 healthcare providers and 15 to 30 intervention group women will provide data on feasibility and acceptability of the intervention. Results of this trial will determine the feasibility and effectiveness of an integrated approach to prenatal mental healthcare and the use of highly accessible computer-based psychosocial assessment and CBT on maternal, infant, and family-based outcomes.

**Trial registration:**

ClinicalTrials.gov Identifier: NCT01901796

## Background

### Prenatal mental health problems

Depression, anxiety, and stress are common in pregnancy. One in four pregnant women experiences symptoms of depression, stress, or anxiety, with 25% having mild to moderate symptoms [1]. Without treatment, up to 48% of women with *prenatal* anxiety and 70% of those with *prenatal* depression [2] continue to experience symptoms through the postpartum period [3-5] and into their children’s early years of life [6-8]. The consequences of poor perinatal mental health are enduring. Two decades of well-conducted longitudinal studies demonstrate that even mild to moderate perinatal distress can have serious adverse effects on mothers and children, including preterm birth and low birth weight [9], child developmental delay [7,10,11], and poor child mental health [12,13].

### The cycle of under detection and under treatment of prenatal depression, anxiety, and stress

To date, perinatal mental healthcare has focused almost exclusively on preventing and treating postpartum depression. This paradigm does not reflect current evidence that 50 to 70% of postpartum anxiety and depression begin [14] and frequently co-occur [15-17] in pregnancy, nor does it reflect the enduring effects of poor prenatal mental health on child health [11,18,19]. Prenatal depression, anxiety, and stress are severely under detected and under treated, and two-thirds of women with substantial symptoms remain unidentified by most obstetrical providers [20,21]. A number of barriers prevent women from seeking mental healthcare during the perinatal period, including stigma, fear of being prescribed medication, lack of knowledge about whether their symptoms are ‘normal’ or ‘abnormal’, and fear that their concerns will be dismissed [22-24]. However, despite recommendations [25,26] and acceptance by both healthcare providers [27-30] and women [31-33], psychosocial assessments are routinely conducted by fewer than 20% of prenatal care providers [34]. In systems without linkages between assessment, referral, and mental healthcare, only 18% of pregnant and postpartum women who are assessed as having mental health problems actually follow up with a referral that they have been given [35], and fewer than 15% of those needing care receive some form of treatment [35,36]. The problem is further complicated by evidence that most women do not voluntarily disclose mental health concerns [22,37,38] (despite the fact that <4% refuse provider-initiated assessment) [39,40]. The cycle of under detection and under treatment is perpetuated by a ‘catch 22’ where providers do not assess women because no follow-up services exist [39], and because women are not assessed, they are not referred and treated. Targeting the individual components of assessment, referral, or treatment in isolation will not address the need in that it is not feasible to enhance psychosocial assessment without simultaneously increasing service capacity to receive referrals. Improvements in psychosocial care can only be addressed as an *integrated* process of assessment-referral-treatment.

### Integrated perinatal mental healthcare

Integrated perinatal mental healthcare - the systematic linkage of assessment, referral, and treatment [41] - has been recommended by national bodies [25]. Integrated care is a more efficient approach to primary care management of depression and anxiety in that it improves access, adherence, and treatment response while being cost-effective [41-44]. Very few studies have evaluated integrated psychosocial care during the perinatal period [40,45,46]. In these studies, the high prenatal ‘screening’ rates of 95% [45] and 62.5% [40] and low refusal rates (<4%) demonstrate women’s acceptance of routine screening and follow-up care [40]. The predominant limitations of existing studies of integrated perinatal mental care (and areas we aim to improve upon) are: 1) all lacked a comparison group; 2) all primarily targeted depression without addressing stress and anxiety; 3) most conducted a minimal feasibility assessment, providing little guidance for improving the intervention or understanding its most effective components; 4) none evaluated clinical outcomes; 5) none used technological (for example, web-based) approaches to support integrated care, although recommended as a key element of success of integrated care [41]; and 6) none targeted the most prominent barriers to mental healthcare reported by providers (for example, lack of time to screen, lack of screening tools and knowledge regarding their use, lack of referral mechanisms, unavailable and inaccessible non-pharmacological therapies) [28,47,48] or by pregnant/postpartum women (lack of time, preference for working through their symptoms on their own, stigma associated with treatment, inability to find/access/afford nonpharmacologic therapy) [22,24,37]. Together, these limitations highlight the lack of utility that current research offers in terms of implementing integrated psychosocial care in clinical settings. There is a need to design and rigorously evaluate integrated interventions that reduce barriers and promote access to mental healthcare by linking standardized psychosocial assessment to effective mental healthcare.

#### Standardized psychosocial assessment

Psychosocial assessment comprises the use of a standardized screening tool (for example, Edinburgh Postnatal Depression Scale, EPDS) in addition to a holistic assessment of psychosocial risk factors (for example, Antenatal Risk Questionnaire, ANRQ-R) [1]. Standardized psychosocial assessment is feasible [31,35,49,50], improves detection [51,52] and facilitates triaging of women by symptom severity to ensure that women receive appropriate services [1]. However, serious resource limitations (for example, lack of time and assessment tools) constrain many primary care providers from routine assessment of mental health problems. Computer-based psychosocial assessment conducted in primary care can address such limitations. Evidence exists that patients and providers find the use of computer-based screening acceptable and feasible for inquiring about sensitive issues, including prenatal [53] and postnatal intimate partner violence [54] and mental health [55,56]. It is also well-suited for busy clinical settings in that it offers consistency, is resource-sparing, can be tailored to meet the needs of patients, can be used with audio/video for low literacy, easily provides a real-time summary for patients/providers [56,57], achieves similar rates of disclosure to written- or interview-based screening, and is preferred by patients due to its perceived anonymity [56,58,59]. However, a recent systematic review demonstrated that, on its own, assessment is ineffective in preventing or treating depression [60] and others have shown that it does not improve linkage with healthcare in the form of follow-up assessment or treatment [21,61]. Thus, in order for mental healthcare to be effective, psychosocial assessment must be systematically linked to treatment.

#### Cognitive behavioral therapy

Cognitive behavioral therapy (CBT) is a highly effective treatment for depression and anxiety [62,63]. Since prenatal mental health problems are characterized by the co-occurrence of anxiety and depression [3,16,17], CBT (including online CBT) is recommended in national guidelines as an early intervention for improving maternal-child outcomes [25]. Randomized controlled trials (RCTs) of group-based CBT for new mothers [64-68] and pregnant women [69,70] demonstrate that group CBT is acceptable and efficacious in reducing risk and symptoms of postpartum depression [64-68].

However, individual- and group-based CBT are frequently inaccessible by pregnant women due to long wait times (groups often small; number of therapists limited) and expense (that is, often not covered by health insurance) [71]. Barriers that are unique to childbearing families (for example, care of other children) can also hinder sustainability of women’s attendance at individual- and group-based CBT sessions [69,72]. Furthermore, pregnant women with mild and moderate symptoms may not be offered CBT due to resource constraints within the healthcare system that restrict these limited services to women with severe symptoms who present with the greatest need at the current time. Consequently, women with mild and moderate symptoms are underserved. Without treatment, there is evidence that 48% of pregnant women with anxiety and 71% of those with depression continue to experience symptoms throughout the postpartum period [2], with as many as one-third of new mothers experiencing symptoms up to 4 years postpartum [73,74]. As such, the delay in not treating pregnant women with mild or moderate symptom severity can lead to substantial personal, societal, and system costs if their symptoms become chronic or more severe over time [75]. Accessible and available mental healthcare is a priority for this vulnerable population.

Few trials have evaluated CBT in pregnancy [66,69,70,76,77]. Pilot testing of a prenatal workbook-based CBT plus telephone coaching by members of our research team revealed four key findings: 1) pregnant women found the program acceptable and helpful; 2) they wanted CBT earlier in pregnancy; 3) they wanted an online format; and 4) they recommended shorter modules [78]. The proposed trial incorporates these pilot results by using six, 30-minute modules (versus the original three), delivering the intervention early in pregnancy (first and second trimester), and adapting the CBT workbook for online use without the use of a telephone coach.

Online CBT is resource-sparing, clinically and cost-effective, acceptable [79-82], and accessible [79], and has been recommended for treatment of anxiety and depression in primary care [83]. A meta-analysis reported that online CBT produces moderate to large effects, is as effective as face-to-face CBT, and has lower attrition rates (20%) than group-based CBT (40 to 50%) [7,84]. Although not tested in pregnant women, online CBT is an ideal treatment because it can overcome major deterrents to mental healthcare cited by pregnant/postpartum women, including: long wait times [35], inaccessibility [35], lack of time [35], finding childcare [24,85], stigma of attending care [24], and treatment expense [35]. Online CBT satisfies the majority (93%) of distressed women’s preference for self-help [24] and should improve aspects of psychological health (for example, mastery, resilience) related to poor pregnancy outcomes [86]. Importantly, online CBT can be embedded in current delivery systems, creating a sustainable approach to effective perinatal mental healthcare. Finally, evidence exists that online CBT and online CBT plus telephone [87] or email [88] support by a psychologist are equally efficacious in reducing depression and promoting adherence. Thus, online CBT, as a stand-alone intervention, offers a highly cost-effective approach to mental healthcare that is independent of limited human resources.

#### Cost-effectiveness

The cost-effectiveness of integrated perinatal mental healthcare has not been evaluated [68,89]. However, an economic evaluation of the cost of treating postpartum depression demonstrated that public health costs were twice as high in women with postpartum depression compared to those without depression [75]. At a prevalence rate of 25% among childbearing women, prenatal mental health problems pose a substantial economic and human resource burden to the healthcare system. However, widespread implementation of integrated prenatal mental healthcare (even resource-sparing approaches) will require a substantial commitment of resources, and an economic evaluation that considers the individual (maternal, family, child), local (clinic-and community-based), and societal implications of early, prenatal intervention compared to usual prenatal care is essential.

#### Mechanisms of integrated psychosocial care

Integrated prenatal psychosocial care is a complex intervention with several components. We found no studies that described mechanisms by which prenatal intervention led to improved outcomes [90]. As noted in the Medical Research Council Framework for Complex Interventions, without this knowledge it is difficult to define which components (for example, program content, intervention characteristics, method of delivery, assessment approach, referral processes) of an intervention contribute to its impact and should be replicated in other settings. Given the need for widely accessible interventions across a diverse spectrum of perinatal care providers and settings (midwives, nurses/nurse practitioners, family physicians, obstetricians), it is critical to identify the key components of the integrated intervention that contribute to its effectiveness and facilitate successful implementation across settings. In practice, a pregnant woman would complete the brief online psychosocial assessment while waiting for her clinic appointment, and her perinatal provider would access these results in ‘real time’ online (for example, a summary of psychosocial risk plus question responses). A decision-making algorithm would provide guidance on the most appropriate referral options for the provider to discuss with the woman. Thus, a key aspect of this study is to understand what aspects of the intervention enhance or deter from its implementation success and integration into routine clinical practice.

#### Maternal-child outcomes

Strong evidence exists supporting a deleterious, enduring effect of poor prenatal mental health on adverse fetal [91] and child outcomes [11,18]. Two decades of longitudinal research have demonstrated a clear, independent association between maternal prenatal distress and neurodevelopmental outcomes in children [11,18,92] and adolescents [93]. Although well established in animal research, early human studies provide evidence of various biological pathways underlying the link between prenatal distress and infant/child outcomes, including epigenetic mechanisms (that is, fetal DNA methylation, placental gene expression [91,94]), impaired neurogenesis [95], and dysregulation of the fetal hypothalamic-pituitary-adrenal (HPA) axis [96,97]. However, the interplay of influences in the prenatal and postnatal environments and, in particular, the extent of the moderating effect of postnatal intervention on fetal development that has been impacted by prenatal depression, anxiety, or stress is largely unknown. Together, this evidence implies that early prenatal intervention should be explored as a means to interrupting the risk of prenatal distress on infant and child well-being.

Very few studies have evaluated the impact of prenatal CBT on infant outcomes [76,98], and none have determined the influence of integrated perinatal mental healthcare on infant or child well-being, maternal early caregiving practices, or the maternal-child relationship. Furthermore, a recent Cochrane review recommended a RCT to explore the value of integrated prenatal psychosocial care on maternal-child outcomes [99]. This is an important line of inquiry, given that symptoms of prenatal depression, stress, and anxiety tend to continue into the postnatal period, influencing the quality of the child’s postnatal environment [7,8,73]. From a healthcare system and societal perspective, the costs associated with poor pregnancy outcomes are substantial [11,18,75,100-102], and treatment options for postpartum mental health [36] and child developmental problems [103] are severely limited. Thus, there is a need to evaluate whether early prenatal intervention can prevent or lessen the risk of adverse maternal-child outcomes.

### The need for a trial

Improvement in perinatal mental healthcare must begin with an integrated, feasible, effective, and resource-sparing approach to routine prenatal psychosocial assessment that is seamlessly linked to referral and treatment across the perinatal period. A systematic review that is being conducted by our research team on the effectiveness of non-pharmacological prenatal interventions on maternal-child outcomes identified four major gaps in prenatal mental health intervention research involving the lack of evaluation of: 1) the clinical effectiveness of integrated psychosocial assessment, referral, and care; 2) the impact of prenatal interventions on prenatal distress or infant/child outcomes (that is, the main outcome has primarily been postpartum depression); 3) factors that contribute to the effect of prenatal interventions (that is, how they worked); and 4) the cost-effectiveness of prenatal interventions. The current study aims to address these four gaps by determining the clinical- and cost-effectiveness of integrated psychosocial assessment-care-referral compared to usual prenatal care, evaluating process outcomes of integrated psychosocial care, and describing the determinants of effectiveness of integrated psychosocial care.

### Research questions

The research objectives, primary and secondary research questions, and hypotheses associated with the four identified knowledge gaps are described in Tables 1 and 2.

**Table 1:** Primary objective, research question, and hypotheses

**Gap**	**objective**	**Research question**	**Testable hypotheses**
**1**	To compare the clinical effectiveness of integrated psychosocial assessment-care-referral versus usual prenatal care on prenatal depression, anxiety, and stress symptoms	What is the effect of integrated, online psychosocial care delivered in pregnancy to women with low or moderate psychosocial risk on the presence and severity of prenatal depression, anxiety, and stress symptoms at 6 to 8 weeks post-randomization compared to usual prenatal care?	Presence of symptoms: Compared to women in the control group, fewer women in the intervention group will have depression, anxiety, and stress symptoms (for example, be above the established cut-off for the DASS21 and EPDS).
			Severity of symptoms: Women in the intervention group will have lower severity of depression, anxiety, and stress (that is, they will have lower mean scores on the depression, anxiety, and stress subscales) compared to those in the control group.

**Table 2:** Secondary objectives, research questions, and hypotheses

**Gap**	**objective**	**Research question**	**Testable hypotheses**
**1-2**	To compare the clinical effectiveness of integrated psychosocial assessment-care-referral versus usual prenatal care on postnatal mental health, psychosocial resources, infant health, and family health	Compared to usual care, what is the effect of integrated, online psychosocial care delivered in pregnancy on:	Compared to women in the control group, those in the intervention will have significantly:
			-decreased presence and severity of depression, anxiety, and stress symptoms at 12 weeks postpartum;
		…..prenatal and postpartum mental health?	-increased psychosocial resources (self-efficacy, mastery, self-esteem, coping); improved sleep quality; and higher relationship quality at 6 to 8 weeks postrandomization and 3, 6, and 12 months postpartum.
		…infant health?	Infants of women in the intervention group will have significantly higher: 1) 5-minute Apgar scores, 2) birth weight, 3) gestational age, 4) maternal-child attachment, and 5) significantly reduced ‘dysfunctional’ infant behavior compared to the intervention group.
		…family health?	The intervention group will have significantly higher parenting competence and partner relationship quality and significantly lower parenting stress compared to the control group.
	To evaluate process outcomes of integrated psychosocial care	Is integrated psychosocial care more efficient, feasible, and acceptable than usual prenatal care?	**Efficiency:** Compared to the control group, a significantly higher percentage of women in the intervention group will have a psychosocial assessment and receive treatment. The intervention group will have significantly lower percentage of women receiving emergency mental healthcare compared to the control group.
			**Feasibility:** ≥ 90% of providers and women report psychosocial assessment is easily done as a component of routine prenatal care, ≥ 95% of intervention group women will access cognitive behavior therapy modules (CBT) within 2 weeks of psychosocial assessment, ≥ 80% of intervention group will access the CBT modules every 1-2 weeks, ≥75% intervention group will complete all CBT exercises, and intervention group will complete 80-100% modules within 6 to 8 weeks.
			**Acceptability:** ≥ 90% of intervention group women and providers will report tablet-based psychosocial assessment during prenatal care acceptable, > 90% women will report that they could provide ‘honest’ responses, and ≥ 90% of intervention group women and providers will find the CBT modules acceptable.
			**Utility:** ≥ 85% of intervention group will report that the CBT homework exercises were useful, and ≥ 90% of intervention group will report each module as useful.
			**Usability:** ≥ 90% of intervention group will report that the exercises and modules were clear, easy to understand, and easy to navigate around.
			(Note. Targets are based on meta-analyses of adherence and satisfaction rates [44]).
**3**	To describe mechanisms of integrated care	What are the mediators and moderators of the intervention effect?	Psychosocial resources (self-efficacy, mastery, self-esteem, coping), sleep, and relationship quality will mediate the impact of the intervention on maternal, child, and family outcomes; and participant characteristics will moderate the effect (for example, demographics, use of antidepressants).
**4**	To compare the cost-effectiveness of integrated psychosocial care compared to usual care	Is the integrated psychosocial care model cost-effective when compared to usual prenatal care?	The expected incremental cost effectiveness of integrated psychosocial assessment, referral, and targeted cognitive behavioral therapy is cost effective at values of health considered acceptable in the Canadian healthcare system.

## Methods/Design

### Design

**Figure 1:**
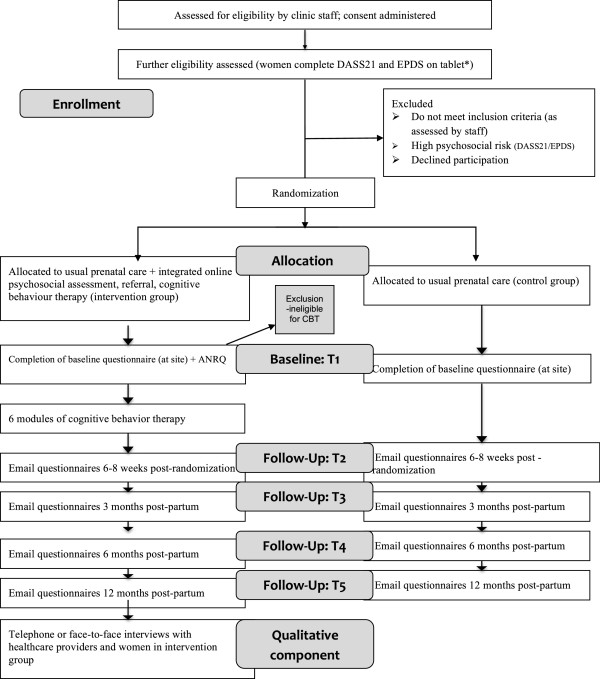
**CONSORT Trial Flow Diagram.** DASS21, Depression, Anxiety, Stress Scale; EPDS, Edinburgh Postnatal Depression Scale.

The proposed study is a randomized, controlled superiority trial of two parallel groups that includes a prospective economic evaluation (Figure 1). It has two phases, including: Phase 1 - a randomized controlled trial designed to evaluate the clinical and cost-effectiveness of an integrated psychosocial assessment-referral-CBT intervention; and Phase 2 - a qualitative descriptive component designed to assess the efficiency, utility, usability, feasibility, acceptability, and mechanisms of the intervention (that is, the active ingredients within the intervention and how they exert their effect) [104]. The research design best suited for answering questions regarding effectiveness, mechanisms, and acceptability/feasibility is a design combining a RCT with a qualitative component [105].

### Randomized controlled trial

#### Setting and recruitment procedures

Recruitment will take place at four primary care clinics in two large, urban Canadian cities. Two of the clinics primarily serve an ethnically diverse, socioeconomically disadvantaged population, with the remaining two clinics serving a largely middle class, Caucasian population. Family physicians in these clinics provide complete and shared prenatal care. Women under complete care receive all prenatal and delivery care from the family physician, while those under shared care receive care from a family physician up to 28 weeks gestation and from an obstetrician thereafter. Family physicians providing care at these clinics do not have specialized mental health training. Eligible women arriving for their prenatal care appointment will be invited to participate in the study by clinic administrative staff. Clinic staff will give women who are interested in study participation a tablet with a link on the main screen to the consent and questionnaire. A research assistant will be available to answer questions about the study. Women agreeing to study participation will complete the electronic consent on a computer tablet.

#### Participant eligibility

Pregnant women are eligible to participate if they are: 1) <28 weeks gestation (Note: The upper limit of <28 weeks allows time to complete six modules and follow-up questionnaires prior to delivery); 2) able to speak/read English; 3) willing to complete email questionnaires; 4) have no, low, or moderate psychosocial risk on screening with the Depression, Anxiety, and Stress Scale at recruitment (Table 3); and 5) are eligible for CBT (Table 3). As in many trials of CBT [68], women on antidepressants will not be excluded because the study objective is not to compare CBT and pharmacological therapy (we plan a subgroup analysis - see Analysis). We are including women with ‘no’ symptoms of psychological distress at the time of recruitment because up to 25% of women in this group may develop mild or moderate symptoms of depression, anxiety, or stress during the course of the trial [106]. In addition, it is important to follow-up subjects who were not identified as having depression, anxiety, or stress symptoms on recruitment in order to understand the implications of false positives (that is, women identified by screening as positive, but who do not have symptoms of depression, anxiety, or stress) and false negatives (that is, women not identified by screening, but who do have symptoms of depression, anxiety, or stress) on the cost-effectiveness of the intervention package as a whole.

**Table 3:** Criteria for ‘high risk’ and referral to physician

**Based on baseline DASS21/EPDS**	**Based on ‘unsuitability’ for CBT (intervention group)**
Women with ‘severe’ or ‘extremely severe’ psychological distress based on one or more of the following criteria:	Women in intervention group with three or more of the following criteria:
1. Depression subscale ≥21 and/or	1. ANRQ-R positive for childhood emotional neglect, childhood emotional abuse, or childhood sexual or physical abuse and/or
2. Anxiety subscale ≥15 and/or	2. ANRQ-R positive for multiple major stressors (for example, major financial issues, bereavement, or separation)
3. Stress subscale ≥26	3. Current substance use or domestic violence
4. EPDS positive Q10 (1, 2, or 3)	4. EPDS positive Q10 or total EPDS score >15

ANRQ-R, Antenatal Risk Questionnaire-Revised; CBT, cognitive behavior therapy; DASS21, Depression, Anxiety, Stress Scale 21; EPDS, Edinburgh Postnatal Depression Scale.

#### Prerandomization

All women will complete the Depression, Anxiety, and Stress Scale (DASS21) and the Edinburgh Postnatal Depression Scale (EPDS) on recruitment (prerandomization) to determine study eligibility and provide baseline data on their mental health status. A computer algorithm designed for this study (Table 3) will calculate symptom scores of the DASS21 and EPDS. If a woman’s scores indicate that she is high risk, a computer-generated message will thank her for her study participation and indicate that the research nurse will contact her. Then, the software program will generate an email to the research nurse who will access the woman’s online assessment results, telephone the woman to inform her that she is excluded from the study, provide feedback on her assessment and, with permission, arrange appropriate referrals.

#### Postrandomization

Women in the intervention group will complete the Antenatal Risk Questionnaire (ANRQ-R). Those who are ‘unsuitable for CBT’ (Table 3) based on the established algorithm using the ANRQ-R and the EPDS will also be excluded from the trial. They will receive a computer message thanking them for their participation and informing them that the research nurse will be contacting them. Within 24 hours, the research nurse will contact these women, inform them of their ineligibility, provide feedback on the ANRQ-R, and, with permission, set up a referral with their healthcare provider. The research nurse will document all referrals made in a computer-based tracking system developed and tested through previous screening trials.

As part of the safety protocol, women in the control and intervention groups assessed as having high symptom scores at any follow-up assessment based on the DASS21 and EPDS will be referred by the research nurse to their healthcare provider (Table 3). All participants assessed as high risk at a follow-up assessment will remain in the trial for the purpose of answering follow-up questionnaires. This is critical to determine whether the integrated intervention facilitates linkage to mental healthcare. Given the stability of untreated mental health symptoms across time [2], we anticipate that an extremely small proportion (4 to 10%) of participants will become high risk after initially being assessed as ‘no’ or ‘low to moderate’ risk at recruitment [14,31].

#### Randomization and allocation procedures

Once eligible women complete the consent to participate on the tablet, a simple computer random number generation algorithm (1:1 allocation ratio) designed for the study by the Clinical Research Informatics Core at the Women’s and Children’s Health Research Institute (University of Alberta) will automatically randomize women. This will be followed immediately by a computer-generated message notifying women of their group assignment. The computer randomization ensures that the research assistant at the clinic is unaware of the participant’s group assignment prior to allocation, and thus allocation concealment is maintained.

#### Sample size estimation

The sample size calculation is based on the primary outcome of symptoms of depression, anxiety, and stress as measured by the Depression, Anxiety, and Stress subscales of the DASS21 [107]. We calculated the sample size required to test the minimum clinically important difference in each subscale and selected the highest one for the final sample size. Based on DASS21 data collected as part of Australia’s national perinatal mental health initiative, standard deviations for the depression, anxiety, and stress subscales in pregnant women are 5.4, 10.2, and 8.6 [108]. To determine the minimal clinically important difference, we used Milgrom et al.’s [66] approach for calculating the difference in scores on each subscale that would shift a woman *one level of severity* - the minimal, reasonable expectation for an effective therapy. For example, the DASS21 manual ‘categorizes’ women as having normal, mild, moderate, severe, and extremely severe symptoms of depression, anxiety, and stress [107]. To shift women from ‘mid-range’ moderate to mild severity on the depression, anxiety, and stress subscales would require a reduction of 4 points in each subscale. Therefore, based on the sample size formula for comparison of two means (2-tailed) at a significance level of 5% (1.96), a power of 80% (.84), and a minimally clinically important difference of 4 points, 204 women with mild to moderate symptoms of psychological distress are required in the trial (Table 4).

**Table 4:** Sample size estimation

N = 2(0.84 + 1.96)^2^ * (**σ**/§)^2^		
σ = standard deviation of the primary outcome (Depression, Anxiety, Stress subscales of DASS21)		
§ = minimal clinically important difference		
Depression subscale	Anxiety subscale	Stress subscale
N = 2(0.84 + 1.96)^2^ * (σ/§)^2^	N = 2(0.84 + 1.96)^2^ * (σ/§)^2^	N = 2(0.84 + 1.96)^2^ * (σ/§)^2^
N = 2(0.84 + 1.96)^2^ * (5.4/4)^2^	N = 2(0.84 + 1.96)^2^ *(10.2/4)^2^	N = 2(0.84 + 1.96)^2^ * (8.6/4)^2^
N = 28.6 per group	N = 102 per group^a^	N = 72.5 per group

^a^Largest sample size per group = 102. Total sample of women with mild to moderate psychological distress = 204. Based on a prevalence rate of 25% of low-moderate symptoms in pregnant women, a final sample size of 816 (408 per group) will be required (204/N = 25/100).

Based on a 25% prevalence rate of low-moderate prenatal psychological distress [1,31], a final sample size of 816 eligible women (408 per group) would be needed. This corresponds to a moderate effect size (d = 0.4 to 0.7) across subscales, which is consistent with a meta-analysis of effect sizes of online CBT [84]. This sample size is also adequate to conduct structural equation modeling to address secondary objectives related to mechanisms underlying the impact of the intervention. Kline recommends a minimum sample size of 200 for complex structural equation models or 10 to 20 cases per level of a variable [109]. Therefore, this final sample size of 816 participants is adequate to address primary and secondary research questions. Accounting for a participation rate of 50% based on previous studies of CBT in pregnant women [110], the exclusion of 15% of women who do not meet study criteria (5% high psychosocial risk [1,31], 10% non-English speaking women), a conservative attrition rate of 35% based on previous studies of prenatal CBT [84], and a 5% loss-to-follow-up (no data reported but our questionnaire follow-up through email should be largely unaffected by change in residence etcetera), 1,673 women would need to be invited to participate in the study to achieve the final sample size. Given that the number of new pregnant patients across the four recruitment sites is 120 per month, the duration of recruitment is anticipated to be 14 months.

#### Intervention

The intervention consists of usual prenatal care plus an integrated intervention comprising: 1) online psychosocial assessment, 2) referral, and 3) online CBT.

#### Online psychosocial assessment

Following randomization to the intervention group, participants will begin the intervention by self-completing the psychosocial assessment (ANRQ-R) on the tablet while waiting for their prenatal appointment. Developed and psychometrically tested by one of our team members [31] the ANRQ-R was designed to be embedded within an integrated system of assessment-referral-care to identify psychosocial risk factors associated with poor mental health outcomes in pregnant women. A system for categorizing level of risk and tailored referral has been devised to enable triaging of women to appropriate services (including CBT) [1]. Its recommended use alongside the EPDS permits identification of psychosocial risk *and* current symptoms (past 7 days). Both instruments can be completed together in less than 10 minutes. The ANRQ-R has high levels of acceptability and satisfactory psychometric properties (sensitivity 0.62; specificity 0.64) [1,31], comparable to other commonly used self-report depression/anxiety tools. The EPDS is a widely used 10-item self-report depression scale used to detect depression symptoms during the previous 7 days [111]. Psychometrically validated for use in pregnant and postpartum women [112], testing revealed sound psychometric properties (sensitivity 86.7%; specificity 78%; positive predictive value 74%, α = .87) [111]. An introductory section to the ANRQ-R describes the importance of routine psychosocial assessment and how it will be used as an initial step to help women with emotional health concerns.

#### Referral

Once women in the intervention group submit their psychosocial assessment, a software program developed for this study will use a scoring algorithm to determine whether the intervention group participant meets criteria for CBT based on ANRQ-R and EPDS scores (see Participant Eligibility). A trained research nurse will receive notification of participant enrolment and telephone all women in the intervention group within 48 hours to review the results of the psychosocial assessment. A standardized approach will be utilized to review the results; however, this review will be tailored to participants’ specific needs in response to their questions and concerns. The research nurse will refer women who meet criteria to the CBT modules (for example, give them the password, web-link, and instructions on accessing and completing the modules).

Women in the intervention group who were assessed as ‘no’ risk on recruitment, but who convert to low or moderate risk at the 6 to 8 week postrandomization follow-up will also be referred to the CBT modules. In this case, the research nurse will receive an automatically generated email and will follow-up with a discussion on symptom scores and referral to the CBT modules. Including women in the study with a gestational age of less than 28 weeks gives sufficient time for women detected at 6 to 8 weeks postrandomization to complete the intervention during pregnancy.

#### Online cognitive behavior therapy

Eligible women in the intervention group will be asked to complete the six, 30-minute online, interactive CBT modules over 6 to 8 weeks [113,114]. Four [77] to six [69] CBT sessions have been found to effectively reduce depression symptoms. The topics of the modules are: 1) taking stock; 2) identifying and labeling emotional health concerns; 3) changing distorted thinking; 4) understanding and changing actions, responses, and behavior; 5) relaxation; and 6) developing and maintaining a plan. Each module has interactive homework assignments that women complete online. Each assignment has one to four options and women select the one (or more) that best suits their needs. Completion of the homework is required before progression in the modules can occur. The modules utilize pregnancy-relevant scenarios and these are used as the basis of examples in the homework assignments. The online delivery allows women to set their own pace by completing the modules at a time and location that is most convenient and ensures standardization of the intervention. Women will access the modules using a username and password, and content that women provide in the homework assignments is accessible only by them.

#### Comparator: usual prenatal care

The control group will receive usual prenatal care. Usual prenatal care at the study sites does not include routine psychosocial assessment or follow-up of psychosocial concerns. Given that this typifies standard prenatal care in the majority of perinatal settings in North America, ‘usual prenatal care’ is the best comparator. All women in the control group will be followed up at 6 to 8 weeks postrandomization and at 3, 6, and 12 months postpartum using the same questionnaires as those delivered to the intervention group. An automatic email will be generated to the research nurse for women in the control group who convert from no, low, or moderate risk to high risk at the 6 to 8 week postrandomization follow-up. The research nurse will contact these women and, with permission, set up a referral to their healthcare provider. These women will continue in the trial to complete all follow-up questionnaires.

#### Definition and measurement of outcomes

##### Primary outcome

The primary outcome is the presence and severity of current prenatal depression, anxiety, and stress symptoms as measured by the DASS21 [107] (Table 5). The DASS21 has been widely used and psychometrically tested; it distinguishes well between symptoms of depression, anxiety, and stress in clinical and non-clinical populations [107,115,116]. It is used in clinical settings to screen pregnant and postpartum women for presence and severity of current symptoms of depression, anxiety, and stress [108,117]. The DASS21 has good psychometric properties with Cronbach α’s of 0.91, 0.80, and 0.84 respectively for depression, anxiety, and stress subscales [116]. High correlations with other standardized depression, stress, and anxiety measures (for example, Beck Depression Inventory, State-Trait Anxiety) and clinical assessments demonstrate its validity [118,119].

**Table 5:** Data collection schedule and measures

**Variable (Measure)**	**Timing of measures**				
	**Baseline**	**6 to 8 weeks post-randomization (pregnancy)**	**3 months postpartum**	**6 months postpartum**	**12 months postpartum**
PHASE I					
Demographics (education, income, maternal age at recruitment, ethnicity) (Items from Maternity Experiences Survey, ^b^MES [120])	**X**				
Obstetric and medical history (parity, chronic and pregnancy complications, type of delivery, weight - pre-pregnancy, delivery, 6 weeks postpartum) (Items from MES)	**X**		**X**		
Mental health history (history of depression, anxiety, stress; age of onset of previous episodes of mental health problems) (Items from MES)	**X**				
Pharmacologic therapy for depression/anxiety (past; current) (Items from Canadian Community Health Survey, CCHS)	**X**	**X**	**X**	**X**	**X**
Social support (Interpersonal Support Evaluation List, ISEL [121])	**X**	**X**	**X**	**X**	**X**
Prenatal depression, anxiety, stress symptoms (Depression, Anxiety, and Stress Scale, DASS-21 [107] - presence (percent above cut-off point) and severity (mean score, standard deviation)	**X**	**X**			
Postnatal depression, anxiety, stress symptoms (Depression, Anxiety, and Stress Scale, DASS-21 [107] - presence (percent above cut-off point) and severity (mean score, standard deviation)	**X**	**X**	**X**	**X**	**X**
^a^Psychosocial assessment (Antenatal Risk Questionnaire-Revised, ANRQ-R; includes substance use and violence) [1,31]	**X**	**X**	**X**	**X**	**X**
Depression (Edinburgh Postnatal Depression Scale, EPDS) [111]	**X**	**X**	**X**	**X**	**X**
^a^ANRQ-R acceptability	**X**				
Mastery (Pearlin’s Mastery Scale) [122]	**X**	**X**	**X**	**X**	**X**
Self-efficacy (Generalized Self-Efficacy Scale) [123]	**X**	**X**	**X**	**X**	**X**
Self-esteem [124]	**X**	**X**	**X**	**X**	**X**
Resilience (Connor-Davidson Resilience Scale) [91]	**X**	**X**	**X**	**X**	**X**
Sleep (Pittsburgh Sleep Quality Index) [125,126]	**X**	**X**	**X**	**X**	**X**
Parenting competence (Parenting Sense of Competence Scale, PSCS; subscales Efficacy, Interest, Satisfaction) [127]			**X**	**X**	**X**
Parenting stress (Parental Stress Scale) [128]			**X**	**X**	**X**
Relationship quality and adjustment (Dyadic Adjustment Scale, DAS-7) [7,129]	**X**	**X**	**X**	**X**	**X**
Coping (Brief Cope) [130]	**X**	**X**	**X**	**X**	**X**
Maternal-infant attachment (Condon and Corkindale) [131]			**X**	**X**	**X**
Infant behavior (Infant Behavior Questionnaire) [132]			**X**	**X**	**X**
Infant development (Ages and Stages Questionnaire, 3^rd^ edition, ASQ-3; The Baby Pediatric Symptom Checklist for Social/Emotional Screening) [91,133]			**X**	**X**	**X**
Birth weight (medical record)			**X**		
Gestational age (medical record)			**X**		
5-minute Apgar score (medical record)			**X**		
Other factors related to infant outcomes: feeding method (medical record and parent-report); neonatal/infant health (medical record and parent-report) (Parent report items from the All Our Babies birth cohort study^c^)			**X**	**X**	**X**
Patient diaries [134] (For economic analysis - including health service use, medication use, productivity loss, personal cost)	**X**	**X**	**X**	**X**	**X**
Quality of life (For economic analysis - SF-36,SF-6D to calculate QALY) [135]	**X**	**X**	**X**	**X**	**X**
Efficiency of intervention (percent of women with psychosocial assessment, referral, and care in IG versus CG; self-report and medical record)			**X**	**X**	**X**
Utility of intervention (one question asked at the end of each cognitive behavior therapy (CBT) homework exercise: *This exercise was useful to me* with four response options of I strongly agree, I somewhat agree, I somewhat disagree, I strongly agree; one question asked at the end of each CBT module: *The information in this module was useful to me* with same response options)		**X**			
Usability of intervention (one question asked at the end of each CBT homework exercise: *This exercise was clear and easy to understand* with response options; 2 questions asked at the end of each module: 1) *The information in this module was clear and easy to understand*; 2) *It was easy to work through the module (for example, it was easy for me to get from one part to the other, easy to find what I needed*) with same response options)		**X**			
Acceptability: Tablet-based psychosocial assessment (one question at end of completing ANRQ-R: *I would recommend a tablet-based approach to asking about emotional health to a pregnant friend* with four response options of I strongly agree, I somewhat agree, I somewhat disagree, I strongly agree)	**X**				
Acceptability: CBT (one question at end of each CBT module: *I would recommend this module to a pregnant friend who was struggling with stress, depression, or anxiety* with 4 response options of I strongly agree, I somewhat agree, I somewhat disagree, I strongly agree)		**X**			
Overall assessment (two open-ended questions at the end of every CBT module: 1) *The thing I liked most about this module was*….; 2) *The thing I liked least about this module was…*.)		**X**			
Log of interactions with participants (completed by research nurse)	**X**	**X**	**X**		
PHASE 2					
Efficiency (Providers’ views of the efficiency of the process of clinic-based online psychosocial assessment)			**X**		
Utility (Women’s views of how useful the modules in were in meeting their needs)					
Usability (Women’s views of how easy/difficult the modules were to navigate)			**X**		
Feasibility (providers’ views of feasibility of conducting integrated intervention in their setting; women’s views of the feasibility of doing the modules; Google Analytics for example, percent of women accessing CBT within 2 weeks postassessment; percent of women accessing each CBT module within 1 to 2 weeks; percent completion of all six CBT modules; percent completion of CBT modules within 8 weeks)			**X**		
Acceptability (women’s and providers’ views of acceptability/ability to promote disclosure)			**X**		
Mechanisms (women’s views of why and how the intervention did/did not improve outcomes; how the intervention benefitted/did not benefit them)			**X**		

^a^Intervention group.

^b^The Maternity Experiences Survey (MES) is a national survey designed and administered by the Public Health Agency of Canada and Statistics Canada. The survey was designed through an exhaustive process involving discussion, consultation, literature reviews, focus group testing, and two pilot studies [120].

^c^The ‘All Our Babies Birth Cohort’ study is a pregnancy birth cohort in Alberta, Canada. Details of the study methodology and design have been previously published [136].

The presence of symptoms of prenatal depression, anxiety, and stress is measured as the proportion of women scoring above established cut-offs (>10; >8; >15, respectively) [107]. Severity of symptoms is measured by the mean depression, anxiety, and stress scores. Ranges of scores corresponding to symptom severity levels of ‘no’, ‘mild’, ‘moderate’, and ‘severe’ are also well-established through psychometric testing: depression (none 0-9; mild 10-13; moderate 14-20; severe > 21); anxiety (none 0-7; mild 8-9; moderate 10-14; severe > 15); and stress (none 0-14; mild 15-18; moderate 19-25; severe >26) [107].

##### Secondary outcomes

All secondary outcomes and their measures are described in Table 5. The secondary clinical outcomes are: presence and severity of symptoms of postpartum depression, anxiety, and stress [107]; prenatal and postnatal self-efficacy [123], social support [121], sense of mastery [122], self-esteem [124], sleep [125,126], relationship quality [7,129], coping [130], and resilience [91]; 5-minute Apgar score; gestational age; birth weight; maternal-infant attachment [131]; infant behavior [132]; infant development [91,133]; and parenting stress/competence [127,128] (Table 5). These outcomes were selected because of their association with maternal depression, anxiety, and stress and their potential modifiability by the intervention. Other secondary process outcomes related to the intervention include its: cost-effectiveness; efficiency; utility; usability; acceptability, and mediators and moderators of effect (Table 5).

#### Data collection

##### Procedures

The five data collection points for all study participants are: recruitment; 6 to 8 weeks postrandomization; and 3, 6, and 12 months postpartum (Table 6). On recruitment, all consent and baseline data are collected via a computer tablet while women wait for their prenatal appointment. Follow-up questionnaires will be completed online. Participants will receive an email with a password and link to the questionnaire on the project website (http://www.yourhope.ca). Retention will be enhanced using Dillman’s approach [137], where women who have not completed the questionnaires within 1 week will receive computer-generated email/smartphone reminders at 1, 3, 7, 10, and 14 weeks by *Checkbox Survey Server*. We will track reasons for nonadherence (for example, lost to follow-up).

**Table 6:** Schedule of enrollment, interventions, and assessments

	**Enrollment**	**Allocation**	**CBT Suitability**	**Post-randomization**				
**TIME POINT**	***-t***_***1***_	**0**	**0**	***T***_***1***__***(Baseline)***_	***T***_***2***__***(6 to 8 weeks postrandomization)***_	***T***_***3***__***(3 months postpartum)***_	***T***_***4***__***(6 months postpartum)***_	***T***_***5***__***(12 months postpartum)***_
**ENROLLMENT:**								
Eligibility screen (based on DASS21 and EPDS)	X							
Informed consent	X							
Allocation		X						
Determination of suitability for CBT (based on ANRQ-R)			X					
**INTERVENTIONS:**								
Psychosocial assessment (ANRQ-R)				X				
Referral				X				
Online cognitive behavior therapy				X	X			
**ASSESSMENTS:**								
Baseline variables^a^				X				
Primary outcome: Depression, anxiety, stress symptoms	X			X	X			
Secondary outcomes -maternal^b^				X	X			
Secondary outcomes -maternal and infant^c^						X	X	X
Utility, usability, acceptability of intervention					X			
Phase 2: Qualitative interviews					X	X	X	

ANRQ-R, Antenatal Risk Questionnaire; CBT, cognitive behavior therapy; DASS21, Depression, Anxiety, Stress Scale; EPDS, Edinburgh Postnatal Depression Scale.

^a^Baseline variables: demographics; history-obstetric, medical, mental health diagnosis and treatment; social support; mastery; self-efficacy; resilience; sleep; partner relationship; coping.

^b^Secondary outcomes - maternal: mental health treatment; social support; mastery; self-efficacy; resilience; sleep; partner relationship; coping; mental health service utilization.

^c^Secondary outcomes - maternal and infant: mental health treatment; social support; mastery; self-efficacy; resilience; sleep; partner relationship; coping; parenting competence; parenting stress; maternal-infant attachment; infant behavior; infant development; gestational age; birth weight; 5-minute Apgar Score; mental health service utilization; quality of life.

##### Management

No data are stored on the tablets; rather, when women ‘submit’ their information it is sent to a secure server housed in the Faculty of Medicine < Dentistry’s Data Centre (University of Alberta). Data transfer between the tablet and server will be encrypted. Follow-up questionnaires will be distributed and submitted via email that is also encrypted. All processes involving electronic data capture and storage are managed by the Women’s and Children’s Health Research Institute Informatics Core at the University of Alberta. Once data collection has been completed, de-identified data will be stored in the Health Research Data Repository at the University of Alberta. Access to the Repository is restricted to research team members conducting analyses.

##### Attrition, adherence, fidelity, and concomitant care

Attrition rates in online CBT are roughly half those [84] of group-based CBT [68]. We will compare attrition rates in the intervention and control groups and conduct telephone interviews with women who drop out of the intervention group to assess reasons. Adherence, that is, the extent to which women complete the psychosocial assessment and CBT components, will be documented through Google Analytics and application specific analytics developed for this study (for example, number modules completed, length of time to complete modules, etcetera). As part of the qualitative descriptive component we will seek women’s opinions about aspects of the psychosocial assessment that were challenging and features of the CBT modules that impacted their ability, need, or desire to complete them.

To improve adherence of women in the intervention group, the research nurse will outline the importance of regular progress through the module homework and the benefit of completing all modules when she provides instructions on how to access the modules. A second key strategy to optimize adherence is the use of email and smartphone reminders to complete the CBT modules if women have been inactive on the site for more than 2 weeks. These reminders will automatically be generated by the software program.

The online format of the intervention preserves its fidelity (that is, consistency in its components and delivery) and thus enhances external validity. To limit co-intervention bias, all women will be discouraged from participating in other self-referred forms of non-pharmacological mental healthcare. However, if the blinded physician detects symptoms of psychological distress in the course of usual prenatal care, the study participant may initiate the recommended pharmacological or non-pharmacological therapy. Follow-up questionnaires will ask women to disclose any pharmacological or non-pharmacological therapy that they have begun and this additional intervention will be accounted for in the analyses.

#### Minimizing the risk of bias

##### Blinding

We will ask women not to share their study involvement with their physician in order to maintain physician blinding and limit the possibility that the physician would change his/her approach to ‘usual prenatal care’. If physicians show greater vigilance in their routine prenatal care as a result of clinic trial involvement, we anticipate that both control and intervention groups would be affected. It is not possible to blind participants due to the nature of the intervention. Women will self-report on all outcomes with the exception of birth weight, gestational age, neonatal health and feeding method at birth, and Apgar scores. These data will be abstracted from the medical record by a research assistant blinded to group allocation, thereby limiting ascertainment bias. Given that current mental health may impact women’s perceptions of infant development and maternal-infant attachment, we will control for *current* mental health when analyzing these outcomes (for example, DASS21 assesses symptoms of depression, anxiety, stress past 7 days). Researchers will be blinded throughout the trial. Finally, the database used for analysis will not include women’s allocation assignment, and therefore the researcher and assistant conducting data analyses will be unaware of group assignment.

##### Selection bias

Selection bias will be limited by consecutive recruitment of women that allows every eligible woman to have an opportunity to participate in the study; however, selection bias will still be a potential factor affecting external validity given the non-random selection of our study sites and the exclusion of non-English speaking women. We also aim to limit threats to internal validity by reducing attrition through the design of the easily accessible, online CBT program rather than a group program. Information bias is minimized due to the use of standardized tools and prospective data collection. Co-intervention may occur if women in the intervention group seek additional formal or informal help for depression/anxiety symptoms in addition to the intervention. We will measure additional service use to parse out the independent effect of the CBT component (for example, subgroup analysis) and to inform the economic analysis. Contamination will be minimized by: 1) having all eligible women ‘do the same thing’ in the clinic (for example, use computer tablet); 2) delivering the intervention away from the physician office; 3) encouraging women not to discuss trial involvement with other patients in the clinic; and 4) ensuring that only women allocated to the intervention group access the CBT modules by having a password-protected entry.

#### Ethics considerations

The study protocol was approved by the Human Research Ethics Board at the University of Alberta. Following electronic consent, all women and sites will receive an emailed copy of the Participant Information Letter and Consent.

#### Safety protocol

Several strategies have been implemented to monitor both intervention and control group women’s psychosocial risk and ensure their safety throughout the trial.

##### Intervention group

Mental health crisis contact information is visible on a sidebar of the online CBT modules and remains accessible at all times. The sidebar also contains a statement encouraging women who feel worse than when they started the intervention to contact the research team’s mental health nurse through a dedicated email link. This message indicates that research nurse will contact the woman within 24 hours. An algorithm will guide the research nurse’s decisions regarding the level of help or referral (for example, to a mental health expert on the research team or woman’s physician) that is provided. Women who were assessed as ‘no’ risk but convert to low or moderate risk at the 6- to 8-week postrandomization follow-up will be contacted by the research nurse and referred to the CBT modules (see Referral). All interactions and decisions will be documented in a computer-based tracking system by the research nurse. The mental health therapists and psychiatrists on the team are available for consultation.

At the end of each CBT module, women will complete question 10 of the EPDS (self-harm thoughts over past week) as a ‘required’ response. An affirmative response (Q10 = 1, 2, or 3) will generate an automatic ‘pop-up’ message with crisis contact information for the woman’s immediate use and an email to the research nurse. The research nurse will contact the woman within 24 hours to assess further and ensure that the woman is receiving help from a healthcare provider. If not, the research nurse will link her to additional resources as guided by an algorithm devised for this study. A 4% affirmative response rate to Q10 of the EPDS has been reported [14]. These women will continue in the trial. A log of interactions and decisions will be maintained by the research nurse.

##### Intervention and control group

For women who convert from no, low or moderate risk (on recruitment) to ‘high’ risk (based on DASS21 and EPDS scores) at any follow-up point, an automatic email will be generated to the research nurse. The research nurse will contact the woman, describe her assessment results, and create a referral to her healthcare provider with her permission. These women will remain in the trial to complete the follow-up questionnaires. All interactions and decisions will be documented in a log by the research nurse.

##### Training

The mental health research nurse will attend a 4-hour training session conducted by mental health experts on the research team regarding the use of the algorithm to guide decision-making and referrals, the availability of local mental health services, and techniques for assisting women in crisis, including domestic violence. The research nurse will collaborate with the perinatal provider and relevant local agencies to provide support.

#### Analyses

##### Effectiveness of intervention

We will use descriptive data (frequencies and 95% confidence intervals, CI; means and standard deviation, SD) to describe the sample. We will test for differences in baseline characteristics using t-tests (means) and chi-squared tests (%). We will assess differences proportions and mean scores of primary and secondary outcomes at each follow-up point using chi-square and t-tests, respectively. We will use an intention-to-treat analysis. We will also use multivariable logistic regression to determine predictors of outcomes and report relative risks and 95% CIs. Multivariable regression models will be built using variables that are associated with outcomes at *P* <0.10 on unadjusted analyses. Primary analyses will use a type I error of 5% as a criterion for statistical significance, while a more stringent alpha of 0.01 will be used for secondary outcomes to account for multiple testing. Because women will be starting the intervention at different points in pregnancy we will control for gestation. We plan to conduct an exploratory analysis using stratified analyses to explore a priori subgroup differences of intervention effect by: (a) number CBT sessions, (b) antidepressant use, (c) symptom clusters, (d) severity of symptoms of psychological distress (DASS21), (e) additional mental health service use, (f) participant characteristics, (g) mental health history, and (h) gestation at time of recruitment. We expect the volume of missing questionnaire data to be low due to the design of the electronic data capture that requires data fields to be populated prior to progressing to subsequent questions. As such, we do not plan to conduct imputation of missing data.

##### Efficiency, utility, usability, and acceptability of intervention

In addition to analyzing the qualitative data that are gathered through Phase 2 interviews, we will use descriptive statistics (frequencies, proportions, means, standard deviations) to describe the efficiency of the intervention (for example, percent of women with psychosocial assessment, referral, and care in intervention group versus control group) and intervention group women’s perceptions of the utility (for example, rated usefulness of exercises and module), usability (for example, rated ease of use of exercises and module), and acceptability (rated willingness to recommend intervention) of the intervention (Table 3). We will also identify the main predictors of these intervention features through multivariable logistic regression and will include variables such as demographic characteristics (including parity), comfort with computer technology, current DASS21 scores, history of mental health problems, and use of ancillary mental health services. All independent variables that are related to each feature at *P* <0.10 will meet criteria for entry to the multivariable models. Adjusted odds ratios and 95% confidence intervals will be reported.

##### Mechanisms of effect of intervention

We will determine factors that influence acceptability and uptake of the intervention using multivariable logistic regression, as adjusted relative risks and 95% CIs. As a preliminary step to model building, we will conduct unadjusted logistic regressions with the criterion for entry into the final multivariable model being *P* <0.05. We will use structural equation modeling (SEM) to describe the direct and mediated effects of the intervention on outcomes. SEM is highly useful for describing complex pathways between an intervention and outcomes that can inform how the intervention has its effect. Consistent with rigorous SEM methodology [109], we will develop a priori models. We will analyze and refine the fit of the model based on recommended model fit indices (for example, model chi-square, Bentler comparative fit index) and theoretical plausibility of the pathways [109]. We will use maximum likelihood estimation for estimation of means, variances, and covariances in order to retain records with missing data in our analysis. We will also analyze qualitative data regarding participants’ and providers’ perspectives on *why* and *how* the intervention did/did not improve outcomes (Table 3).

##### Cost-effectiveness of intervention

The economic evaluation will be a within-trial cost effectiveness analysis comparing the integrated intervention ‘package’ with usual prenatal care. The perspective of the primary analysis will be that of the health and social care budget. A secondary analysis will adopt a societal perspective incorporating personal costs and productivity costs in addition to the health and social costs associated with the delivery of the intervention (for example, cost of equipment, salary of research nurse and clinic staff) and subsequent service utilization by study participants. Direct healthcare utilization will be extracted from patient records. Patient Diaries will be completed at 3-month intervals from randomization to end-of-follow-up, to gather retrospective accounts of other health and social care utilization, out-of-pocket expenses and productivity costs (Table 5). The SF-36 will be completed at the same time points as the Patient Diaries. The Patient Diaries will contain validated resource utilization and productivity cost questionnaires such as PRODISQ [134]. The primary outcome measure for the cost effectiveness analysis will be the Quality Adjusted Life Year (QALY). Utilities for the construction of QALYs will be obtained from the SF-36 data using the SF-6D algorithm [135]. As the time horizon for the analysis is less than 12 months, discounting will not be required [138]. We will report the incremental cost per QALY gained for the integrated intervention compared to usual prenatal care. Uncertainty in the expected costs and outcomes for the integrated intervention and usual prenatal care will be characterized using the non-parametric bootstrap. The results of the bootstrap analysis will be used to construct scatterplots on the cost effectiveness plane and cost-effectiveness acceptability curves showing the probability that the integrated intervention is a cost effective use of healthcare resources for a range of values of health.

### Qualitative descriptive study

#### Design

Incorporating participant perspectives and experiences to assess the suitability and utility of interventions is critical in trials of complex interventions in order to identify components that may influence the outcomes [139]. Phase 2 utilizes a qualitative descriptive study to assess women’s and healthcare providers’ views on efficiency, utility, usability, feasibility, acceptability, and potential mechanisms of action of the intervention.

#### Methods

##### Participant eligibility and recruitment

All intervention group participants and healthcare providers working at study sites are eligible for participation in Phase 2. Purposeful sampling will be used to maximize variability in the sample, ensuring that a broad range of views and demographics are represented [140]. We plan to interview 15 to 20 intervention group women and 8 to 10 providers (for example, nurses, family physicians) with the final sample size determined by data saturation. Given the importance of understanding factors contributing to attrition, we will also interview intervention group women who do not complete all CBT modules. In order to capture women who may not complete all six modules, a notification at the end of each of the fourth, fifth, and sixth CBT modules will invite women in the intervention group to participate in a follow-up interview. Selection of the affirmative response will generate an automatic email to the research coordinator for follow-up. Emails distributed by each of the clinic managers will invite clinic staff members to participate in a follow-up interview.

##### Data collection and management

We will conduct individual face-to-face or telephone-based interviews. Semi-structured interview guides will be used [140] to ask participants their views on the efficiency, utility, usability, feasibility, acceptability, and mechanisms of action of the intervention (Table 3), as well as its strengths, suggestions for improvement, components that were effective/not effective, and the benefits that they experienced. Interviews are expected to take one hour and will be digitally recorded and transcribed verbatim. Transcribed interviews and digital files will be password protected and stored on a password protected computer in a secure, locked office. Digital files will be stored for 5 years and then deleted. All data will be anonymized for publication.

##### Analysis

As recommended for qualitative descriptive studies, we will use standard qualitative content analysis approaches for thematic analysis of the transcripts [140]. Two members of the team experienced in qualitative data analysis will independently code the first two or three transcripts, discuss, and reach consensus on a preliminary coding scheme. This coding scheme will be applied in the coding of another two to three transcripts, following which the two researchers will discuss a revised coding scheme. At this point, the coding scheme will be sufficiently developed to allow one research team member to independently code the remaining transcripts, with revisions made as necessary to reflect new and evolving themes as data analysis progresses [140]. Thematic analysis will occur concurrently with data collection to allow further exploration and clarification of emergent ideas, and data collection will continue until data saturation is reached [141].

### Trial status

The IMPACT: Pilot and IMPACT: RCT trials were funded within a few months of each other. Recruitment for the IMPACT: RCT has been deferred to February, 2014 to permit collection of pilot data. The pilot and full RCT share the same study design and methodology, with the exception that the pilot study will collect data to 3 months postpartum, and the full RCT will collect data to 12 months postpartum. The same recruitment sites will be utilized for both the pilot and full RCT. Data from the pilot trial will be used to refine the intervention, the economic evaluation, and the logistics involved in the full trial. At the time of manuscript submission, the CBT modules and the online version of the psychosocial assessment component (ANRQ-R and EPDS) are being finalized and recruitment for IMPACT: Pilot will begin in September, 2013. Trial registration is through ClinicalTrials.gov (Identifier: NCT01901796).

## Abbreviations

ANRQ-R: Antenatal Risk Questionnaire; CBT: cognitive behavioral therapy; DASS21: Depression, Anxiety, and Stress Scale; EPDS: Edinburgh Postnatal Depression Scale; IMPACT: Integrated Maternal Psychosocial Assessment to Care Trial; QALY: Quality Adjusted Life Year; RCT: randomized controlled trial; SEM: structural equation modeling.

## Competing interests

The authors declare that they have no competing interests.

## Authors’ contributions

DK conceived and designed the study, drafted the grant and the protocol manuscript, organizes and supervises trial implementation, and is responsible for trial management, staff training and supervision. MPA, AB, KH, GL, SM, SDM, and SVZ participated in writing the grant. MPA, AB, KH, GL, WS, MH, SM, SDM, and SVZ contributed to the study design. KH, GL, SDM, TP, and SVZ participated in study implementation. MLS manages day-to-day trial responsibilities, including supervising staff, monitoring recruitment and data collection, and liaising with recruitment sites. SVZ provides expertise on study methodology and advises on trial management. MPA, KH, and GL provide mental health expertise, AB, MH and SDM provide obstetrical expertise, and SM and RG provide child development expertise. SM provides statistical and methodological expertise and DK, SM, and RG will conduct statistical analyses. MLS, DK, KH, and GL will conduct qualitative interviews and DK, GL, KH, MLS, MH and WS will analyze qualitative data. All authors contributed to the development of the CBT modules. All authors participated in refinement of the study methods, critically reviewed manuscript drafts, and approved the final manuscript.

## Authors’ information

DK (PhD) is an Assistant Professor in the Faculty of Nursing and an Adjunct Assistant Professor in the Department of Obstetrics and Gynecology at the University of Alberta. She holds an Early Career Transition Award through the Alberta Centre for Child, Family, and Community Research. MPA (MD, FRANZCP, MB) is a perinatal psychiatrist and Professor in the Faculty of Medicine at University of New South Wales. She is also the Chair of the Perinatal and Women’s Mental Health Unit at the University of New South Wales, the Director of the St. John of God Mother-Baby Unit in Sydney, Australia, and the lead developer of the *Australian Clinical Guidelines for Perinatal Mental Health* (2011) and the International Marce Society Position Statement on *Psychosocial Assessment and Depression Screening in the Perinatal Period* (2013). AB (MD, CCFP, FCFP) is a family physician in the Mount Sinai Academic Family Health Team and an Associate Professor in the Department of Family and Community Medicine at the University of Toronto. She holds the Ada Slaight and Slaight Family Directorship in Maternity Care in the Ray D. Wolfe Department of Family Medicine at Mount Sinai Hospital in Toronto. RG (PhD) is a Senior Research Fellow and Clinical Psychologist at the Murdoch Children's Research Institute, Melbourne, Australia. MH (PhD) is a Professor in the Faculty of Nursing and an Associate Professor in the Departments of Community Health Sciences and Obstetrics, Gynecology and Reproductive Sciences at the University of Manitoba. She held a CIHR Chair in Gender and Health award from 2008-2013. KMH (PhD) is a Professor in the Faculty of Nursing and an Adjunct Professor in the Department of Psychiatry at the University of Alberta. She holds a Canada Research Chair in Stress Disorders in Women. GL (PhD) is an Associate Professor in the Faculty of Nursing at the University of Alberta and is a Certified Psychiatric Nurse. SM (PhD) is an epidemiologist with expertise in life course analysis, mental health tool development, and child development. She is the senior scientist for the All Our Babies birth cohort study. SDM (MD, FRSCS, MSc) is an Associate Professor in the Division of Maternal-Fetal Medicine in the Departments of Obstetrics and Gynecology, Radiology, and Clinical Epidemiology and Biostatistics. She holds a CIHR New Investigator Award. TP (MD, CCFP, MSc) is a family physician and an Assistant Professor in the Department of Family Medicine at McMaster University. She is the Co-Head of Service in Family Medicine Obstetrics and the Hamilton Site Coordinator of the Behaviour Science Program. WS (PhD) is a Professor in the School of Nursing at McMaster University. MLS (MA) is the Research Coordinator for the HOPE (Healthy Outcomes of Pregnancy and Postpartum Experiences) Program of research. SVZ (MD, PhD) is Director of the Division of Gastroenterology at University of Alberta Hospital and a trial methodologist.
